# Impact of epicardial adipose tissue volume upon left ventricular dysfunction in patients with mild-to-moderate aortic stenosis: A post-hoc analysis

**DOI:** 10.1371/journal.pone.0229636

**Published:** 2020-03-02

**Authors:** F. Hardt, M. Becker, V. Brandenburg, J. Grebe, T. Dirrichs, R. F. Gohmann, K. Fehrenbacher, J. Schmoee, S. D. Reinartz

**Affiliations:** 1 Department of Diagnostic and Interventional Radiology, University Hospital RWTH Aachen, Aachen, Germany; 2 Department of Cardiology, Angiology and Intensive Care, University Hospital RWTH Aachen, Aachen, Germany; University of Dundee, UNITED KINGDOM

## Abstract

**Background:**

Aortic stenosis (AS) may lead to diastolic dysfunction and later on heart failure (HF) with preserved left ventricular ejection fraction (HFpEF) via increased afterload and left-ventricular (LV) hypertrophy. Since epicardial adipose tissue (EAT) is a metabolically active fat depot that is adjacent to the myocardium and can influence cardiomyocytes and LV function via secretion of proinflammatory cytokines, we hypothesized that high amounts of EAT, as assessed by computed tomography (CT), may aggravate the development and severity of LV hypertrophy and diastolic dysfunction in the context of AS.

**Methods:**

We studied 50 patients (mean age 71 ± 9 years; 9 women) in this preliminary study with mild or moderate AS and mild to severe LV diastolic dysfunction (LVDD), diagnosed by echocardiography, who underwent non-contrast cardiac CT and echocardiography. EAT parameters were measured on 2nd generation dual source CT. Conventional two-dimensional echocardiography and Tissue Doppler Imaging (TDI) was performed to assess LV function and to derive myocardial straining parameter. All patients had a preserved LV ejection fraction > 50%. Data was analysed using Pearson’s correlation.

**Results:**

Only weak correlation was found between EAT volume or density and E/é ratio as LVDD marker (r = -.113 p = .433 and r = .260, p = .068 respectively). Also, EAT volume or density were independent from Global Strain Parameters (r = 0.058 p = .688 and r = -0.207 p = .239). E/é ratio was strongly associated with LVDD (r = .761 p≤0.0001) and Strain Parameters were moderately associated with LV Ejection Fraction (r = -.669 p≤0.001 and r = -.454 P≤0.005).

**Conclusions:**

In this preliminary study in patients with AS, the EAT volume and density as assessed by CT correlated only weakly with LVDD, as expressed by the commonly used E/é ratio, and with LV strain function. Hence, measuring EAT volume and density may neither contribute to the prediction nor upon the severity of LVDD, respectively.

## Introduction

Heart failure (HF) is distinguishable into systolic heart failure (SHF) with an impaired LV ejection fraction or diastolic heart failure (DHF) with impaired filling of the left ventricle [1]. In DHF the impaired filling due to diastolic dysfunction and underlying structural heart disease is not easily measurable by a unique parameter or modality but can be described by a bouquet of conditions. In the absence of a comprehensive understanding of the mechanisms of this illness, DHF is also referred to as HF with preserved LV ejection fraction (HFpEF) as a practical definition. By this definition about half of all cases of HF are represented and due to the aging population, its prevalence is on the rise [2, 3]. HFpEF is a common reason for hospital admission and is associated with age, arterial hypertension, obesity and diabetes [4–6]. The diagnosis of HFpEF relies on signs and symptoms of HF and can be described by the degree and presence of LV diastolic dysfunction (LVDD) [7]. LVDD can be measured by invasive laevocardiography or echocardiography as a reliable non-invasive method [8]. But the interpretation of Doppler variables in relation to patient age and clinical setting can be difficult due to interobserver variabilities, potentially leading to different estimates of diastolic dysfunction [9]. In the course of HF accompanying comorbidities such as diabetes, renal failure or chronic obstructive pulmonary disease the illness can worsen over time and lead to a poorer prognosis. Therapeutic options against HFpEF are limited, since no treatment has successfully reduced mortality so far [10]. Hence, it is mandatory to obtain better insights into risk factors, pathophysiology and concomitant diseases of this condition.

Aortic Stenosis (AS), as the most common valvular lesion in the western world [11], impairs left-ventricular function via increased afterload. Elevated LV systolic pressure leads to concentric LV hypertrophy with wall thickening, which results in diastolic dysfunction due to decreased ventricular compliance and impaired early diastolic relaxation [7, 12]. LV hypertrophy due to AS or arterial hypertension as a form of underlying heart disease is a major contributor to impaired LV filling and HFpEF.

Myocardial Deformation (Strain Imaging) analysis is an application of echocardiography to quantify LV function and enables for quantifying LV dysfunction. There are two options for Strain Imaging, tissue doppler derived strain imaging (TDI) or 2D speckle tracking echocardiography (STE). Both have been validated for myocardial deformation analysis [13]. In this study, we used TDI for strain analysis. Longitudinal Strains has been validated as a reliable tool to stratify cardiovascular prognosis in some cardiac disorders e.g. HF and is more sensitive than LV EF in detecting LV systolic dysfunction [14].

Epicardial adipose tissue (EAT) is a metabolically active fat depot that makes up for approximately 20% of total heart weight and lies on top of the myocardium and in the interatrial grooves around the coronary arteries [15, 16]. EAT consists of mainly adipocytes, but also entails ganglia, connecting nerves and immune cells [17]. EAT is strongly associated with obesity, metabolic syndrome and coronary artery disease (CAD) [18]. EAT can influence cardiomyocytes and cardiac function via a secretion of proinflammatory adipokines [17, 19].

The finding of a previous study that EAT is associated with the deterioration of diastolic function over time, fuells our hypothesis of possible disadvantageous local myocardial effects of EAT [20, 21], which potentially makes EAT an universal diagnostic and stratification tool for LVDD. However, this data was obtained in the absence of AS, hence it remains obscure if EAT plays a similar role regarding LVDD in this valvular disease where increased afterload is a confounding major driver of LVDD, too. Therefore, we investigated the association of EAT with LVDD in patients primarily selected via the presence of mild to moderate AS in this preliminary study.

## Methods

### Study population

This study is a post-hoc analysis of an interventional prospective single-center study (URL: http://www.clinicaltrials.gov. Unique identifier: NCT00785109; reviewed and approved by RWTH Aachen Institutional Review Board No. 165/08), where we retrospectively derived EAT parameters from CT datasets and included echocardiographic studies which measured LVDD and myocardial deformation (Strain) [22]. Patient enrolment took place between January 2010 and August 2015. Written, informed consent was obtained for the prospective study. For evaluation of the secondary outcome measurement (“development of diastolic and systolic dysfunction”), this post-hoc analysis was performed.

In this cross-sectional study we selected from the 99 patients of the entire study group patients with a LV EF of > 50% and echocardiographic presence of mild or moderate aortic stenosis. A total of 50 patients (age 71 ± 9 years; 8 women) were included into the posthoc analysis. Most important exclusion criteria were history of Coronary Artery Bypass Graft Surgery (CABG) and non-completion of CT-graphic and echocardiographic studies. Patients were recruited irrespective of a history of coronary artery disease. Patients underwent a non-contrast cardiac CT for quantification of valvular and vascular calcification and measurement of epicardial adipose tissue volume following departments standards. Known cardiac risk factors were assessed and defined as follows: arterial hypertension was defined as having a systolic blood pressure ≥ 140 mmHg and/or diastolic blood pressure ≥ 90 mmHg or use of antihypertensive drugs. Obesity was defined as a body mass index (BMI) greater than 30 kg/m^2^. Diabetes mellitus was defined as use of oral hypoglycemic agents or insulin. Dyslipidaemia was defined as use of a cholesterol lowering agent.

### CT protocol

CT scans were performed with a second-generation, ECG-synchronized 128-slice dual source computed tomography (DSCT) scanner (SOMATOM, Definition Flash, Siemens). Collimation was 64 x 2 x 2 x 0.6 mm, Voltage was set to 120kV, time current product was referenced to 80mAs. Scan range was from tracheal bifurcation to the diaphragm. 3.0 mm reconstructions with 1.5mm increment and a dedicated kernel (B35f) were used. Besides standardized quantification of coronary calcium scoring (CAC) and quantification of aortic valve calcifications using “Ca-Scoring” (Siemens, Leonardo MMWP), non-contrast cardiac CT is used for detection and quantification of EAT-volume (EATV) based on tissue density differences. For the measurement of EATV, the volume measurement software “Volume” (Leonardo MMWP, Siemens), was used. First the pericardium was identified manually for EAT volume and density quantification in each axial slice. Then, all voxels representing EAT with a density range from -190 to -40 Hounsfield Units [HU] within the pericardium were automatically quantified. Assessment for EATV was done below the pulmonary trunk till the end of the left ventricular apex to standardize the z-axis coverage. Total EATV was acquired by summing up the EAT area of all axial slices, multiplied by the slice thickness [3mm] and the number of slices.

### Echocardiography

The echocardiographic studies were performed by two experienced cardiologists on a Vivid 7 Ultrasound System (GE Vingmed, Horton, Norway). Datasets were acquired for three full cardiac cycles with a frame rate of at least 50 frames/sec at three parasternal short axes [at the Mitral Valve (SAX-MV), at the apical level (SAX-AP) and at the midpapillary level (SAX-PM)] and three apical long axes [2- and 4-chamber views and at the apical level (APLAX)]. The mitral inflow E velocity was acquired by pulsed Doppler and é velocity was averaged from the septal and lateral mitral annuli. Strain Parameters were acquired in the endocardial, midmyocardial and the epicardial layer and averaged. Analysis of TDI images was performed offline with a customized software (Echopac, GE Medical Systems) by two independent observers. Evaluation of LV Diastolic Dysfunction was done in accordance with present guidelines and recommendations [9, 10].

### Statistical analysis

The statistical analysis was performed using IBM SPSS Statistics 23.0 (SPSS, Chicago, IL, USA). All continuous parameters are expressed as means ± standard deviations or n (%) where applicable. The distribution of the data was checked for normality using the Shapiro-Wilk test. Then the correlation between EAT parameters and indices of cardiac function assessed by TDI was evaluated with Pearson’s correlation. A 2-tailed p-value of <0.05 was set for statistical significance.

## Results

### Baseline characteristics

50 Patients with a mean age of 71 ± 9 years were included in this study. Of those, 42 (84%) were male. 39 (78%) had arterial hypertension, 8 (16%) had diabetes mellitus, 25 (50%) had dyslipidaemia, 26 (52%) had coronary artery disease and 6 (12%) were active smokers. Mean observed body weight was 81 ± 13 kg, the average BMI in the study population was 27 ± 4 kg/m^2^ (range: 19–36 kg/m^2^) and 16 (32%) were obese (BMI ≥ 30 kg/m^2^). For more details please refer to Table 1.

**Table 1:** Baseline characteristics of the study population.

Category	Parameter	
**Demographics**	Age, [years]	71 ± 9
	Male Sex, [n, %]	42 (84)
	Body Weight, [kg]	81 ± 13
	BSA, [m^2^]	1.9 ± 0.2
	BMI, [kg/m^2^]	27 ± 4
	Duration of disease (aortic stenosis), [month]	7.00 ± 10.2
**Risk factors**	Obesity, [n, %]	16 (32)
	Hypertension, [n, %]	39 (78)
	Diabetes, [n, %]	8 (16)
	Known coronary artery disease, [n, %]	26 (52)
	Smoking, [n, %]	6 (12)
	Dyslipidemia, [n, %]	25 (50)
	LDL, [mg/dL]	115.26 ± 36.5
	HDL, [mg/dL]	54.32 ± 14.2
	Total-Cholesterol, [mg/dL]	192.78 ± 40.2
	Triglycerides, [mg/dL]	145.47 ± 83.9
**Vital signs**	Heart rate [bpm]	71.38 ± 16.1
	normal blood pressure, [n, %]	42 (84%)
**Medication**	Aspirin, [n, %]	36 (72%)
	Clopidogrel, [n, %]	11 (22%)
	ACE inhibitor, [n, %]	26 (52%)
	β-Blocker, [n, %]	36 (72%)
	Diuretics, [n, %]	22 (44%)
	Calcium Antagonist, [n, %]	9 (18%)
	Statin, [n, %]	32 (64%)
	Alpha-Blocker, [n, %]	3 (6%)
	Antidiabetic drug, [n, %]	7 (14%)

all summary values are mean ± standard deviation or n (%).

### CT findings

For all participants, mean EAT volume was 128.40 ± 47.14 [cm^3^]. Mean EAT density was -84.69 ± 14.46 [HU] and the mean noise index of EAT density was 25.48 ± 16.33 [HU]. EAT volume index [EATV], EAT volume normalised on body surface area [BSA], was 65.64 ± 21.95 [cm^3^/m^2^]. Male participants had a mean EAT volume of 129.60 ± 49.41 [cm^3^] and mean EAT density was -83.77 ± 15.48 [HU]. For female participants, EAT volume was 122.14 ± 34.63 [cm^3^] and mean EAT density was -89.55 ± 5.40 [HU].

### Echocardiographic findings

Echocardiographic datasets of diagnostic quality could be obtained for all patients. Mean LV ejection fraction for all patients was 60.72 ± 7.45 [%]. Measurements at height of the aortic valve, as seen in Table 2, were as follows: Aortic Valve Peak velocity was 2.81 ± 0.53 [m/s], mean pressure gradient was 17.81 ± 6.78 [mmHg] and aortic valve area was 1.34 ± 0.30 [cm^2^].

**Table 2:** CT-graphic and echocardiographic findings.

EAT Volume (EATV) [cm^3^]	128.40 ± 47.14
EAT Density [HU]	(-) 84.69 ± 14.46
EATV Index [cm^3^/m^2^]	65.64 ± 21.95
EF [%]	60.72 ± 7.45
GLS [%]	(-) 18.12 ± 3.90
GCS [%]	(-) 19.86 ± 4.55
E/e´ Ratio	13.5 ± 4.5
MV DT [ms]	239 ± 49
LVDD [%]	48 (96)
Mild LVDD [%]	10 (20)
Moderate LVDD [%]	22 (44)
Severe LVDD [%]	16 (32)
Aortic Valve (AV) V max [m/s]	2.81 ± 0.53
AVA [cm^2^]	1.34 ± 0.30
AV Δp [mmHg]	17.81 ± 6.78

MV DT, mitral valve deceleration time; LVDD, left ventricular diastolic dysfunction; AVA, aortic valve area; Δp, pressure gradient; all summary values are mean ± standard deviation or n (%).

Global strain at the long axis for the endocardial layer was -20.98 ± 4.60 [%], -18.01 ± 3.87 [%] at the midmyocardial layer and -15.53 ± 3.38 (%) for the epicardial layer. Hence, averaged Global Longitudinal Strain (GLS) was -18.12 ± 3.90 [%]. Global strain at the short axis for the endocardial layer was -28.88 ± 8.01 (%), for the midmyocardial layer -18.56 ± 4.31 (%) and -12.14 ± 2.58 (%) for the epicardial layer. Therefore, mean Global Circumferential Strain (GCS) was -19.86 ± 4.55 [%]. Measurements at mitral valve for evaluating LV diastolic dysfunction are included in Table 2. LV diastolic dysfunction was present in 48 (96%) patients. 10 patients (20%) had mild LVDD, moderate LVDD was present in 22 patients (44%) and 16 patients (32%) had severe LVDD.

### Correlation of EAT, strain parameters, LV dysfunction and cardiovascular risk factors

**Fig 1:**
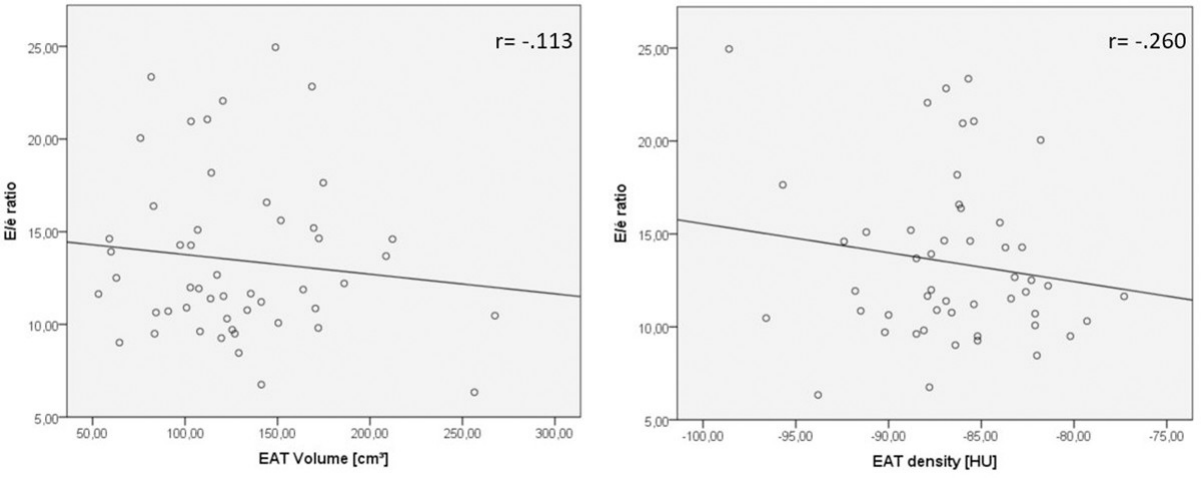
Correlation between EAT volume & density and E/é ratio. Scatterplots for the weak correlation between EAT volume and EAT density with E/é ratio (r = -.113, p = .433 or r = -.260, p = .068).

**Fig 2:**
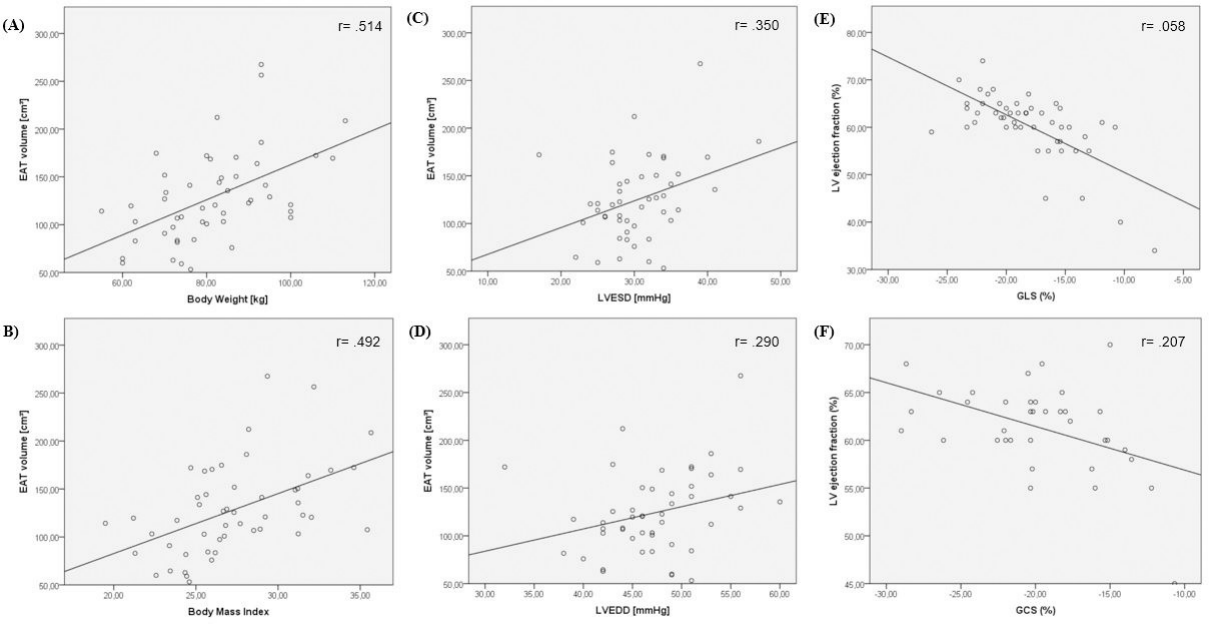
Correlation between EAT volume and body weight, BMI, LV pressure & LV EF and GCS, GLS. Correlation scatterplot between EAT volume and (A) body weight (r = .514, p = ≤.0001) (B) BMI (r = .492, p = ≤.0001) (C) LV end-systolic pressure (r = .350, p = .016) (D) LV end-diastolic pressure (r = .290, p = .045). Correlation scatterplot between LV ejection fraction and (E) Global Longitudinal Strain (r = .058, p = .688) (F) Global Circumferential Strain (r = .207, p = .239).

Weak correlation was found between EAT volume or density and E/é ratio (r = -.113, p = .433 or r = -.260, p = .068 respectively) (Fig 1). Also, EAT volume was independent of Global Longitudinal Strain (GLS) and Circumferential Strain (GCS) (r = .058, p = .688 and r = .207, p = .239). EAT volume normalized on body surface area (EATV index) also did only yield weak correlations with E/e´ ratio or Global Strain parameters GLS or GCS (for all p = ≥.05). As demonstrated in Table 3, EATV was significantly associated with LV end-diastolic pressure (r = .290, p = .045) and even more so with LV end-systolic pressure (r = .350, p = .016) (both Fig 2). EATV index also correlated with LV end-systolic pressure (r = .317, p = .030). EAT volume was moderately associated with body weight (r = .514, p = ≤.0001) (Fig 2), body mass index (r = .492, p = ≤.0001) and obesity (r = -.477, p = ≤.0001), but not significantly with other measured cardiovascular risk factors as arterial hypertension, diabetes or dyslipidaemia (all p ≥ .05). E/e´ ratio did correlate with EAT noise index (r = .350, p = ≤.013) and was strongly associated with LV diastolic dysfunction (r = .761, p≤.0001). Global strain parameters GLS and GCS are representative for LV EF (r = -.669, p≤.001 and r = -.454, p = .005) (Fig 2).

**Table 3:** Correlation analysis of EAT volume with study parameters.

	Correlation Coefficient	P
E/e´ Ratio	0.133	0.433
Deceleration Time	0.096	0.508
Global Longitudinal Strain	0.058	0.688
Global Circumferential Strain	0.207	0.239
LV Ejection Fraction	0.119	0.410
LV end-diastolic pressure	0.290	0.045
LV end-systolic pressure	0.350	0.016
Age	0.077	0.597
Body Weight	0.514	≤ 0.001
Body Mass Index	0.492	≤ 0.001

## Discussion

Aim of this study was to evaluate diagnostic capability of EAT in patients with mild-moderate AS with LVDD. But, our preliminary data could not establish a reliable connection between the degree of LVDD as assessed by echocardiography (goldstandard) and the amount of EAT as assessed by cardiac CT. Neither did EAT volume or density correlate with known marker of LVDD, i.e. E/e’ ratio, nor did it relate with Strain parameters GLS or GCS.

As an inspiration for our study, recent preliminary data pointed towards a potential association between the amount of EAT and the presence of HFpEF [23] but without AS. Furthermore, they included patients with a wide range of comorbidities associated with diastolic dysfunction. Hence our study expands these findings in a meaningful way by providing data derived in a specifically selected patient cohort at risk for HFpEF, i.e. patients with increased afterload induced by mild to moderate AS. AS as the most common left valvular disease [11] with a combination of changes in LV size, LV wall thickening and fibrosis leads potentially to both changes in systolic and diastolic function.

This retrospective study measured EAT by cardiac MRI in short-axis slices using a commonly used cine technique (Steady-State Free-Precession-SSFP: slice thickness = 6mm, gap = 4mm), extended beyond the atrio-ventricular valves to cover the entire pericardium in endsystole and -diastole. Our CT examinations come as 3D dataset without gaps and a halved slice thickness compared to MRI. Therefore, CT based EAT reading claims to measure more accurately because of the improved epicardial coverage and image resolution and especially since the danger of erroneous inclusion of a pericardial effusion is not present in contrast to MRI examinations. Since EAT values are not changing during heart circle, differentiation between systole/diastole might not be necessary. The functional evaluation as provided by strain analysis via echocardiography (goldstandard) in our study provides comparable results to MRI, too. Myocardial deformation (strain) analysis showed abnormal values for GLS in about 30% of all patients with a cut-off value for GLS ≥ -16% based on existing literature [24, 25]. A higher GCS was previously described for AS [26, 27] as an expression for LV mechanical adjustment to chronically increased afterload. In line with these studies we found, on average, elevated values for both GLS and GCS. In this context, a specific strength of our study is that we provide sensitive parameters for the presence of left ventricular dysfunction by echo strain data, which is more reliable than a simplified classification according to certain ranges of LV-EF [23, 28].

EAT has been shown to produce pro-inflammatory and pro-atherogenic cytokines [16, 17, 29, 30] and its increase in volume has been described for the extent of coronary artery disease, with the degree of coronary artery calcifications and with severe AS [31], but we were not able to support those findings but to demonstrate a close link between higher EAT volume and body weight, body surface area and obesity. Doesch et al. [32] measured EAT volume in patients with dilated cardiomyopathy using MRI and found that it was associated with HF with reduced EF, but not with a preserved EF as in our study. Other studies are in line with that and show a correlation for EAT and reduced LV-EF [33, 34]. We concur that the influence of EAT volume on LVDD in patients with AS might be of minor concern as shown by our preliminary data and that an increase in afterload and other changes associated with AS may play a more important role in causing LVDD and LV hypertrophy. Considering this aspect, increased EAT volume can be interpreted as structural fat depots with minor endocrine functionality in this disease.

In addition to that, Serrano-Ferrer et al. provides in individuals affected by metabolic syndrome a link between EAT and Strain [35]. Cavalcante et al. demonstrated, using CT and TDI measurement methods, a link between EAT volume, LV diastolic dysfunction and E/e’ ratio in healthy overweight individuals [18]. So, there is nevertheless evidence that EAT volume is a factor in the development of LV dysfunction in certain circumstances [20].

Furthermore, within our rather small study population there were no significant differences between genders in association with EAT volume and cardiac function, which seems to be less important in this elderly cohort [6].

However, we could not replicate the suspected correlations between LVDD and EAT in patients with AS, where increased volume and pressure loading seem to be more important driver of LV dysfunction rather than EAT volume alone. We conclude that major changes in EAT volume maybe are only present in advanced AS and HF with a reduced EF, and not in early valvular disease as was the case in our patient cohort [36]. Furthermore, EAT volume or density maybe does not adequately reflect the underlying pathological mechanisms in HFpEF and LVDD, where EAT volume and density may yet not change, but our preliminary data does neither confirm nor reject this assumption. So further studies regarding the development of EAT volume change in different stages of AS are needed.

### Limitations

Our post-hoc study is supposed to be preliminary data and has several limitations: the use of tissue doppler imaging rather than speckle tracking echocardiography for strain rate imaging may have led to a greater variance of straining parameters due to noise and intra- and interobserver variability. Furthermore, our data was incomplete for longitudinal echo parameters and we could not supply any molecular biomarkers. Another aspect to keep in mind when looking at our results is the large proportion of men (84%) within our relatively small sample size (n = 50), in which significant coronary artery disease was not ruled out clinically. Since this is an elderly cohort, gender is statistically less important than age [6]. The one-time measurement of all data prohibits analysis of development of EAT volume change in HF and different stages of LVDD. Hence, data are absent whether the amount of EAT may predict the occurrence of HF with reduced ejection fraction. Lastly, our data do not allow a comprehensive EAT analysis over the entire spectrum of AS.

## Conclusion

In this preliminary study, epicardial adipose tissue volume as assessed by CT has no obvious influence on LVDD, as it does not correlate well with echocardiographic indices of LVDD as E/é ratio in patients affected by mild to moderate AS.

## Supporting information

S1 Table Patient information: CT- and echocardiographic measurements. (PDF)
