# Supplementary material for: Impact of epicardial adipose tissue volume upon left ventricular dysfunction in patients with mild-to-moderate aortic stenosis: A post-hoc analysis
Source: PLoS One. 2020 Mar 2;15(3):e0229636. doi: 10.1371/journal.pone.0229636 (PMC7051069; doi:10.1371/journal.pone.0229636)
Supplement: S1 Table — (PDF) [file pone.0229636.s001.pdf]

S1 Table. Patient information: CT- and echocardiographic measurements

Study ID	EAT volume [cm³]	EAT density [HU]	GLS [%]	GCS [%]	LV EF [%]	LVEDP [mmHg]	LVESP [mmHg]	MV DDT [ms]	E/é	Diastolic Dysfunction
K001	83,51	-85,2	-26,33	-14	59	47	32	255	9,5	1
K004	113,79	-86,9	-10,78	-20,33	60	42	25	237	11,39	1
K005	112,03	-85,4	-13,33	-13,56	58	53	34	186	21,06	3
K008	172,05	-88,1	-22		74	32	17	247	9,81	1
K014	62,84	-82,3	-23,33	-22	60	42	28	234	12,51	2
K017	103,16	-83,7	-23,33	-26,44	65	46	35	290	14,27	2
K022	117,27	-83,2	-13,56		45	39	31	281	12,67	2
K023	97,34	-82,8	-15,44	-16,22	57	45	30	242	14,28	3
K024	102,86	-87,7	-19,33		61	42	29	216	11,99	1
K026	119,62	-85,2	-22,44	-28,33	63	45	27	204	9,26	1
K029	90,88	-82,1	-15,33		60	49	29	252	10,71	2
K030	84,3	-90	-22,22	-28,67	68	51	28	248	10,64	2
K034	120,79	-83,4	-18,33		63	46	25	226	11,52	2
K037	126,91	-80,2	-19	-18,33	63	45	33	186	9,5	3
K041	75,89	-81,8	-17,89	-24,56	64	40	30	180	20,05	3
K043	122,7	-79,3	-15,67	-20,22	57	48	28	172	10,31	1
K058	151,8	-84	-21,11	-19,56	68	51	36	254	15,61	3
K060	103,21	-86	-18,33	-18	63	47	28	253	20,95	3
K063	163,89	-82,6	-10,33		40	53	27	285	11,88	1
K065	186,04	-81,4	-16,67	-10,67	45	53	47	188	12,21	2
K068	174,72	-95,7	-18,11		67	43	27	321	17,64	3
K070	125,52	-90,2	-20	-22,56	60	43	32	280	9,71	2
K074	129,04	-82	-20,56		65	56	34	273	8,46	1
K075	141,24	-87,8	-21,56	-20,5	67	55	28	202	6,75	0
K077	133,65	-86,6	-19,67	-20,17	63	49	28	243	10,77	2
K081	170,49	-91,5	-17,67	-15,33	60	51	34	219	10,86	2
K082	53,18	-77,3	-15,44	-20	64	51	34	231	11,64	2
K083	212,17	-92,4	-15,78		65	44	30	247	14,6	2
K084	82,94	-86,1	-11,89	-22,11	61	46	29	207	16,38	3
K087	59,94	-87,7	-17	-15,67	63	49	32	189	13,92	2
K088	100,8	-87,4	-20	-20,33	64	47	23	250	10,9	2
K092	148,89	-98,6	-24	-15	70	47	31	335	24,95	3
K093	144,14	-86,2	-22	-24,22	65	49	29	282	16,58	3
K094	135,54	-87,9	-23,33	-22	64	60	41	231	11,66	2
K096	120,44	-87,9	-18,78	-26,17	60	46	24	295	22,06	3
K097	108,1	-88,5	-20,89	-20,33	63	44	28	283	9,62	1
K100	107,48	-91,8	-16,11	-29	61	42	26	276	11,93	2
K102	141,21	-85,4	-14,11		55	51	35	271	11,21	2
K103	169,54	-88,8	-17,33	-20,33	55	56	40	400	15,2	3
K106	256,45	-93,8	-15,33		55			191	6,34	0
K107	208,68	-88,5	-16,44	-12,22	55			241	13,68	2
K109	64,52	-86,4	-18,33	-19,33	63	42	22	230	9,02	1
K110	267,56	-96,6	-22,67		61	56	39	223	10,47	2
K111	168,62	-86,9	-19,22	-21,67	60	48	34	219	22,83	3
K113	114,18	-86,3	-7,44		34	48	36	94	18,18	3
K115	106,8	-91,2	-20,22	-17,67	62	44	26	184	15,1	3
K117	172,36	-87	-14,67	-15,17	60	51	32	242	14,64	2
K061	59,05	-85,6	-19,11	-18,22	65	49	25	223	14,62	2
K062	150,41	-82,1	-20,44		62	46	33	172	10,08	2
K112	81,69	-85,7	-13	-16	55	38		258	23,35	3

EAT: epicardial adipose tissue; GLS: Global Longitudinal Strain; GCS: Global Circumferential Strain; LV EF: Left Ventricular Ejection Fraction; LVEDP: Left Ventricular End-Diastolic Pressure; LVESP: Left Ventricular End-Systolic Pressure; MV DDT: Mitral Valve Diastolic Deceleration Time; E/é: ratio between mitral peak velocity of early filling (E) to early diastolic mitral annular velocity (é)
